# Phillygenin Attenuates Carbon Tetrachloride-Induced Liver Fibrosis *via* Modulating Inflammation and Gut Microbiota

**DOI:** 10.3389/fphar.2021.756924

**Published:** 2021-09-21

**Authors:** Cheng Wang, Cheng Ma, Ke Fu, Li-Hong Gong, Ya-Fang Zhang, Hong-Lin Zhou, Yun-Xia Li

**Affiliations:** State Key Laboratory of Southwestern Chinese Medicine Resources, Key Laboratory of Standardization for Chinese Herbal Medicine, Ministry of Education, School of Pharmacy, Chengdu University of Traditional Chinese Medicine, Chengdu, China

**Keywords:** Phillygenin, liver fibrosis, gut microbiota, intestinal barrier, carbon tetrachloride

## Abstract

Liver fibrosis is a chronic pathological process that various pathogenic factors lead to abnormal hyperplasia of hepatic connective tissue, and its main feature is the excessive deposition of extracellular matrix. However, there are currently no drugs approved for the treatment of liver fibrosis. Phillygenin (PHI), a lignan isolated from Forsythiae Fructus, showed potential anti-inflammatory and anti-fibrosis effects but the mechanisms remain unknown. In view of the vital role of gut microbiota in the development of liver fibrosis, this study aimed to explore whether PHI could protect intestinal epithelial barrier and attenuate liver fibrosis by maintaining the homeostasis of intestinal microbiota. Therefore, the liver fibrosis model was induced by intraperitoneal injection of olive oil containing 10% carbon tetrachloride (CCl_4_) for 4 weeks in C57BL/6J mice. Histological analysis including Hematoxylin-Eosin, Masson, Sirius red, and immunohistochemistry staining were carried out to detect the histopathology and collagen deposition of mice liver tissues. The biochemical indexes related to liver function (ALT, AST, AKP, γ-GT), fibrosis (HYP, HAase, LN, PC III, IV-C) and inflammation (TNF-α, MIP-1, LPS) were determined by specific commercial assay kits. *In vivo* experimental results showed that PHI could improve liver histopathological injury, abnormal liver function, collagen deposition, inflammation and fibrosis caused by CCl_4_. Moreover, PHI restored the intestinal epithelial barrier by promoting the expression of intestinal barrier markers, including ZO-1, Occludin and Claudin-1. More importantly, the corrective effect of PHI on the imbalance of gut microbiota was confirmed by sequencing of the 16 S rRNA gene. In particular, PHI treatment enriches the relative abundance of *Lactobacillus*, which is reported to alleviate inflammation and fibrosis of damaged liver. Collectively, PHI attenuates CCl_4_-induced liver fibrosis partly *via* modulating inflammation and gut microbiota.

**GRAPHICAL ABSTRACT:**
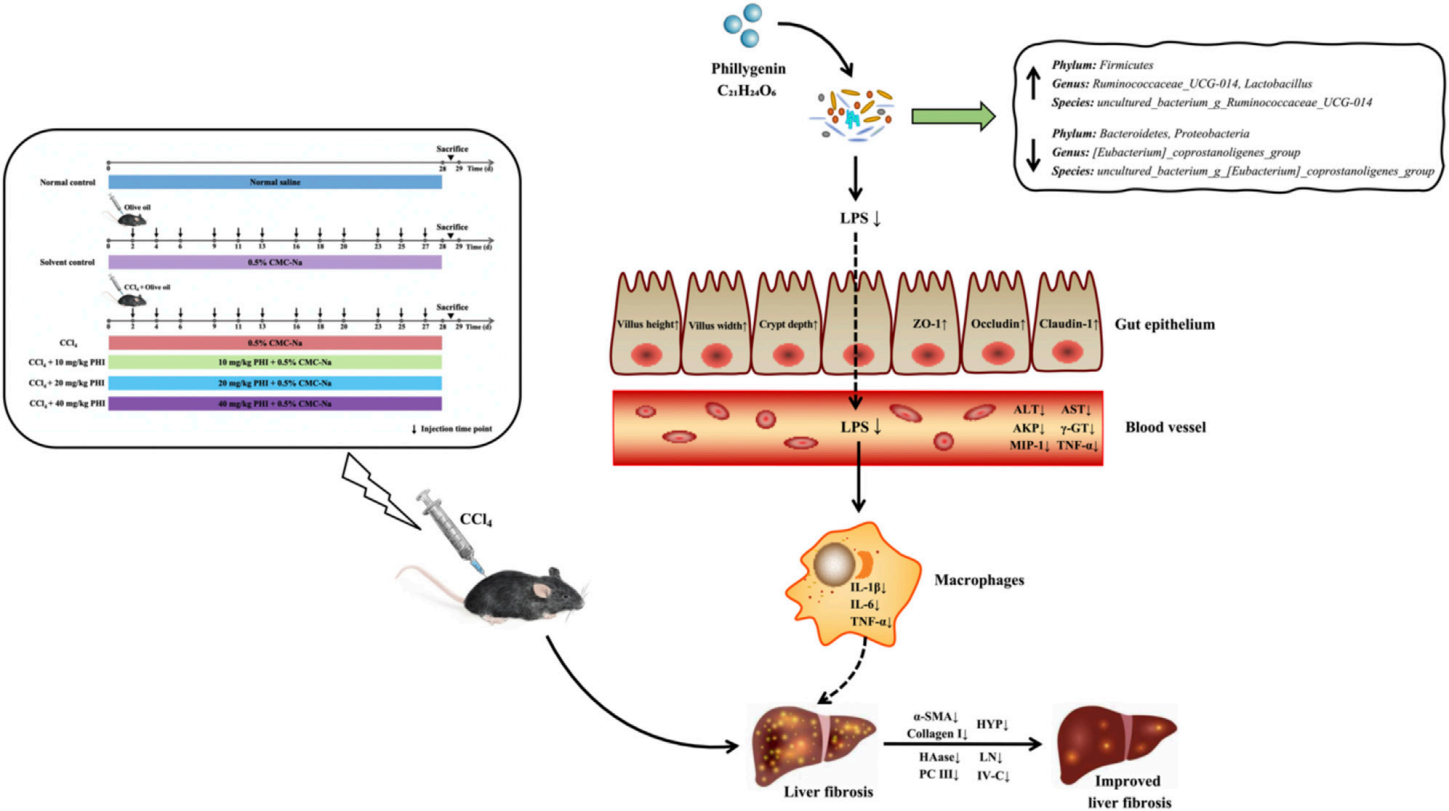


## Introduction

Liver fibrosis is a dynamic pathophysiological process which intrahepatic connective tissue undergoes dysplasia during chronic liver injury caused by various pathogenic agents (Aydın and Akçalı, 2018). Its essence is an excessive reparative response of the liver to tissue injury, which is primarily characterized by excessive deposition of extracellular matrix (ECM) (Tsuchida and Friedman, 2017). Common pathogenic factors include viral hepatitis, chemical drugs or poisons, autoimmune diseases, genetic and metabolic diseases (Campana and Iredale, 2017). If the pathogeny of chronic liver injury is not promptly removed, inflammation and liver fibrosis will be continuously activated, which may further develop into liver cirrhosis or even hepatocellular carcinoma (Parola and Pinzani, 2019). At present, the effective treatment for patients with advanced liver fibrosis or liver cirrhosis is only liver transplantation (Koyama et al., 2016). Fortunately, some studies have shown that liver fibrosis is a reversible pathological feature in the early stage of chronic liver disease (Sun and Kisseleva, 2015). Therefore, it is urgent to develop effective, safe and economical anti-hepatic fibrosis drugs.

In recent years, a large body of literature has shown that intestinal flora disorder plays a vital role in the occurrence and development of anti-liver fibrosis drugs (Zhou et al., 2019; Liu et al., 2021). In brief, changes in the composition of the intestinal microbiota and intestinal permeability cause gastrointestinal disorders, with disruption of the intestinal barrier, and then pathogen-associated molecular patterns such as abnormal bacterial fragments and products enter the liver *via* the portal system, which promote the release of pro-inflammatory cytokines, and in turn accelerate the development of liver fibrosis (Kumar et al., 2011; Zhou et al., 2019). Interestingly, many studies have confirmed that changing the structure of intestinal flora can alleviate the progression of liver fibrosis. For example, dietary fiber can reduce liver fibrosis, partially relying on changing the intestinal flora structure and increasing the Bacteroidetes/Firmicutes ratio (Li et al., 2021). Similarly, Tan et al. (Tan et al., 2020) showed that the mixture of triterpenoid from yeyachun and phenolic acids from danshen could significantly improve CCl_4_-induced liver fibrosis in mice by reducing the production of profibrotic metabolites of intestinal flora.

Forsythiae Fructus, the dried fruit of *Forsythia suspensa* (Thunb.) Vahl, has a wide range of pharmacological effects including anti-inflammation, antioxidant, anti-fibrosis and antibiosis (Zhang Y. et al., 2018; Wang et al., 2018). As an important active ingredient of lignans as well as a fingerprint component in Forsythiae Fructus, phillygenin (PHI) have also been confirmed to have good hepatoprotective effects in recent years (Song et al., 2018). Interestingly, our group previously found that PHI could inhibit lipopolysaccharide-induced pro-inflammatory responses and the activation of LX2 cells through the TLR4/MyD88/NF-κB signaling pathway to alleviate liver fibrosis (Hu et al., 2020). However, the mechanisms of PHI against liver fibrosis are complex and have not been fully elucidated so far. Furthermore, in view of the important role of intestinal microbiota in the development of liver fibrosis, it remains unclear whether the anti-fibrotic effects of PHI are partly dependent on the regulation of intestinal flora disorder. Therefore, in this research, we aimed to determine the ameliorative effect of PHI on intestinal flora disorder in mice with CCl_4_-induced liver fibrosis and explore its potential mechanisms, with a view to developing a promising preventive alternative for CCl_4_-induced liver fibrosis.

## Materials and Methods

### Reagents

PHI (MUST-20060710, purity ≥98%) was purchased from Must Bio-Technology Co., Ltd. (Chengdu, China). Carbon tetrachloride (P1491992) was obtained from Titan Scientific Co., Ltd. (Shanghai, China). Olive oil (2019102901) and Sodium carboxymethyl cellulose (2018022601) were collected from Chron Chemicals Co., Ltd. (Chengdu, China). 4% paraformaldehyde (CR2011054) was purchased from Servicebio Technology Co., Ltd. (Wuhan, China). Animal total RNA isolation kit (R201101) was obtained from Foregene Co., Ltd. (Chengdu, China). ABScript II RT Master Mix (9620041118) and Genious 2X SYBR Green Fast qPCR Mix (9620041114) were purchased from ABclonal Technology Co., Ltd. (Wuhan, China).

### Animal Study

Eight-week-old male C57BL/6J mice (20 ± 2 g) were obtained from Chengdu Dashuo Biotechnology Co., Ltd. (Chengdu, China). All the mice were housed in a standard animal laboratory (23 ± 2°C, relative humidity 50 ± 20%) with a 12-h light/dark cycle. The mice had free access to food and clean water ad libitum. All the experimental procedures were performed according to the Guide for the Care and Use of Laboratory Animals of the National Institutes of Health. The study was approved under the regulations of the Committee on the Ethics of Animal Experiments of Chengdu University of Traditional Chinese Medicine (Permit Number: SCXK 2013-19). All animals were adaptively fed for 7 days before experiments.

**FIGURE 1:**
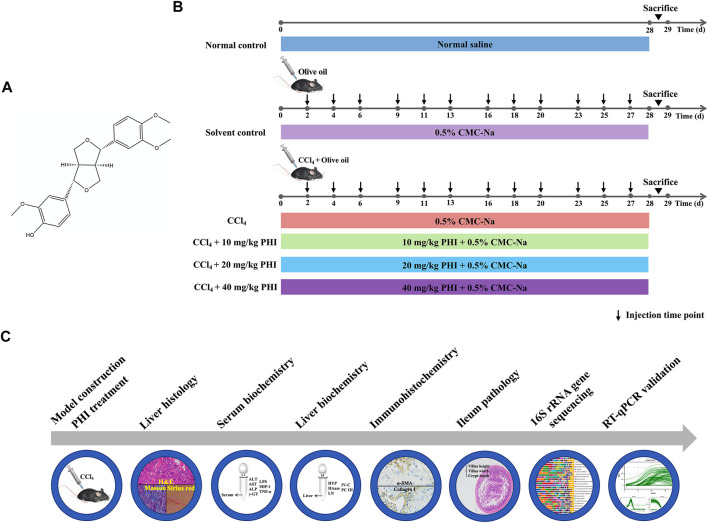
Experimental outline. **(A)** Chemical structure of PHI. **(B)** Flow diagram depicting the treatment of mice in all groups. **(C)** Description of the experimental design.

36 mice were randomly divided into six groups (*n* = 6/group) (Figure 1): (1) Normal control group, (2) Solvent control group, (3) CCl_4_ model group, (4) CCl_4_ + 10 mg/ kg PHI group, (5) CCl_4_ + 20 mg/ kg PHI group, (6) CCl_4_ + 40 mg/ kg PHI group. Except for the normal control group and the solvent control group, mice in the other groups were injected with CCl_4_ dissolved in olive oil (1:9, v/v) three times a week (2 ml/ kg) intraperitoneally (i.p.) for 4 weeks to establish liver fibrosis model. The solvent control mice were injected with olive oil alone at the same volume and frequency. PHI was dissolved in a 0.5% sodium carboxymethyl cellulose (CMC-Na) solution. Mice were given PHI (intragastric administration, i. g.) every day for 4 weeks. Normal control groups were given normal saline to minimize the effects of the gavage procedure. Solvent control groups were given 0.5% CMC-Na to eliminate the influence of solvent. At the end of the trial, all the animals were euthanized. The serum was collected. The liver, colon and ileum tissues were excised and weighed, and a part of the tissues were fixed in 4% paraformaldehyde. The rest of the tissues were preserved at -80°C refrigerator for further analysis.

### Hematoxylin and Eosin Staining

Liver tissues and intestinal tissues (ileum) were fixed with 4% paraformaldehyde for 24 h, dehydrated and embedded in paraffin. All other processes were performed in accordance with standard procedures. 4 μm sections were cut and stained with H and E for pathological analysis. Furthermore, the villus height, villus width and crypt depth of ileum were measured by Image-pro plus 6.0 software.

### Serum Biochemical Index Detection

Serum was collected from blood samples after centrifugation for 15 min at 2000 × g and 4°C. Serum alanine aminotransferase (ALT), aspartate aminotransferase (AST), alkaline phosphatase (ALP) and γ-glutamyl transpeptidase (γ-GT) levels were measured with specific commercial assay kits according to the manufacturer’s instructions. ALT test kit (20210407) and AST test kit (20210406) were purchased from Nanjing Jiancheng Bioengineering Institute (Nanjing, China). ALP colorimetric assay kit (76DVRRBQ13) and γ-GT colorimetric assay kit (YNPLPSE6PS) were obtained from Elabscience Biotechnology Co., Ltd. (Wuhan, China). In addition, the serum levels of lipopolysaccharides (LPS), macrophage inflammatory protein 1 (MIP-1) and tumor necrosis factor α (TNF-α) were detected in accordance with the instructions of enzyme-linked immunosorbent assay (ELISA) kits. Mouse LPS ELISA kit (20191122) and Mouse MIP-1 ELISA kit (202103) were obtained from Meimian industrial Co., Ltd. (Jiangsu, China). Mouse TNF-α ELISA kit (GBANGISHML) was obtained from Elabscience Biotechnology Co., Ltd. (Wuhan, China).

### Collagen Deposition Detection

Liver tissues were fixed in 4% paraformaldehyde for 24 h and dehydrated, embedded and sliced. Collagen deposition in the liver was observed by Masson staining and Sirius red staining.

### Liver Biochemical Index Detection

Liver hydroxyproline (HYP) content was determined by alkaline hydrolysis method as described in kits. In addition, the contents of hyaluronic acid (HAase), laminin (LN), collagen type Ⅳ (IV-C) and precollagen type Ⅲ (PC Ⅲ) in liver tissue were detected according to the instructions of ELISA kits. HYP test kit (20210405) was purchased from Nanjing Jiancheng Bioengineering Institute (Nanjing, China). Mouse HAase ELISA kit (202103), Mouse LN ELISA kit (202103), Mouse IV-C ELISA kit (202103) and Mouse PC Ⅲ ELISA kit (202103) were obtained from Meimian industrial Co., Ltd. (Jiangsu, China).

### Immunohistochemistry Staining

Tissue sections, which were 4 μm thick 4% paraformaldehyde-fixed and embedded in paraffin, were prepared as follows. The samples were dewaxed in xylene and rehydrated in alcohol. Subsequently, antigen was retrieved in a full citrate buffer (pH 6.0) and incubated with 3% hydrogen peroxide at room temperature for 25 min in dark. Then, specific antibodies were added and incubated overnight at 4°C. The protein was detected using 3,3′-diaminobenzidine staining. The nucleus was stained by hematoxylin. Finally, α-smooth muscle actin (α-SMA) and collagen I were observed by microscope.

### Gut Microbial Sequencing

Genomic DNA was extracted from the feces of mice using a stool DNA kit (Tiangen biochemistry, China) according to the manufacturer’s instructions. The yield and quality of DNAs were measured by micro plate spectrophotometer (GeneCompang Limited, China) and 1.8% agarose gel electrophoresis (Bomei Fuxin, Beijing, China), respectively. Primers (27F 5′-AGRGTTTGATYNTGGCTCAG-3′ and 1492R 5′-TASGGHTACCTTGTTASGACTT-3′) were used to amplify the bacterial full-length region of the 16 S rRNA gene. The sequencing service was provided by Biomarker Technologies Co., Ltd (Beijing, China). Alpha diversity, principal coordinates analysis, species distribution histogram, linear discriminant analysis effect size, functional prediction and correlation heat map were further processed with a bioinformatic pipeline tool, BMK Cloud online (www.biocloud.net). The original 16 S rRNA sequencing data were deposited into the Sequence Read Archive database of NCBI, and the accession number is PRJNA758124 (https://www.ncbi.nlm.nih.gov/bioproject/PRJNA758124).

### Real-Time Quantitative PCR Analysis

Total RNA was extracted from liver and intestinal tissues by using animal total RNA isolation kit. The total RNA extracts were dissolved in 50 μL RNase-free water. The RNA purity was detected with the nucleic acid/protein analyzer by measuring the value of OD260/280.5X ABScript II RT Mix performed reverse transcription for the synthesis of cDNA. Reaction conditions were set as follows: 25°C for 5 min, 42°C for 15 min, 85°C for 5 s and 12°C for hold. The qPCR was performed on the StepOnePlus Real-Time PCR System by adding the Genious 2X SYBR Green Fast qPCR Mix (No ROX) according to the manufacturer’s protocol. Reaction conditions were carried out as follows: 95°C for 3 min followed by 40 cycles at 95°C for 5 s and 60°C for 30 s. The specific sequences of primers used in this study were synthesized by TSINGKE Biological Technology (Chengdu, China) and primers sequences were listed in Table 1. Melting curve analysis was performed at the end of each PCR run to ensure amplification of a single product. The relative mRNA expression levels were calculated by the 2^−ΔΔCT^ method.

**TABLE 1:** Specific primers sequences used in RT-qPCR.

Gene	Forward primer (5′–3′)	Reverse primer (5′–3′)
α-SMA	CTC​TGT​CTG​GAT​CGG​TGG​C	TTC​GTC​GTA​TTC​CTG​TTT​GCT
Collagen I	TGA​CCT​TCC​TGC​GCC​TAA​TG	GCT​ACG​CTG​TTC​TTG​CAG​TG
IL-1β	GAA​GAA​GAG​CCC​ATC​CTC​TG	TCA​TCT​CGG​AGC​CTG​TAG​TG
IL-6	CTG​CAA​GAG​ACT​TCC​ATC​CAG	AGT​GGT​ATA​GAC​AGG​TCT​GTT​GG
TNF-α	GAC​AGT​GAC​CTG​GAC​TGT​GG	TGA​GAC​AGA​GGC​AAC​CTG​AC
ZO-1	GGG​AAA​ACC​CGA​AAC​TGA​TG	GCT​GTA​CTG​TGA​GGG​CAA​CG
Occludin	CCC​AGG​CTT​CTG​GAT​CTA​TGT	TCC​ATC​TTT​CTT​CGG​GTT​TTC​A
Claudin-1	CTG​GGA​TGG​ATC​GGC​TCT​ATC	CCT​CGT​AGA​TGG​CCT​GAG​CA
GAPDH	ATG​GGT​GTG​AAC​CAC​GAG​A	CAG​GGA​TGA​TGT​TCT​GGG​CA

### Statistical Analysis

Data were analyzed using SPSS 25.0 software, and statistical significance was determined by t-tests. Multiple groups were compared using one-way analysis of variance. Results are expressed as means ± SD, and results with p values of less than 0.05 were considered significant. All graphs were generated using Graph Pad Prism (Graph Pad Software, United States).

## Results

### Results of HE Staining and Serum Biochemical Indexes

**FIGURE 2:**
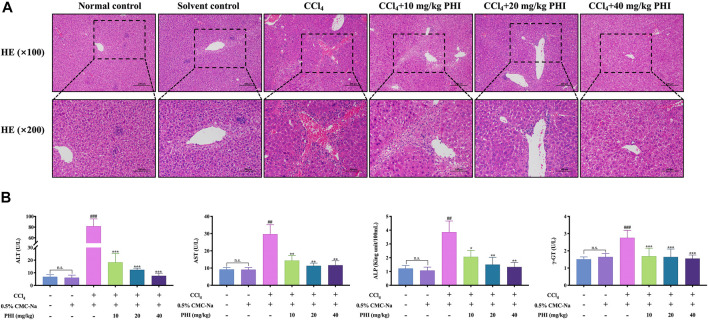
Effect of PHI on the pathological and serum biochemical indexes of liver fibrosis in mice. **(A)** HE staining of liver ( × 100 and × 200). **(B)** Serum ALT, AST, AKP and γ-GT activity. Data was expressed as the mean ± SD (*n* = 6). ^##^ *p* < 0.01, ^###^ *p* < 0.001 represent compared with solvent control group. *p < 0.05, **p < 0.01, ***p < 0.001 represent compared with CCl_4_ group. n. s indicates no significant.

As shown in Figure 2A, HE staining showed that the hepatocytes in the normal control group and solvent control group were closely arranged, and there was no obvious hepatic parenchymal cell lesion and inflammatory cell infiltration. In the CCl_4_-treated mice, significant hepatocyte steatosis, eosinophilic cytoplasmic enhancement and collagen fiber proliferation were observed. Furthermore, there was a small amount of lymphocyte infiltration in the portal area of the lobule. And PHI (10, 20 and 40 mg/ kg) treatment alleviated the pathological changes of liver tissues in different degrees (Supplementary Material).

The results of serum biochemical indexes are shown in Figure 2B. There is no significant difference between the normal control group and the solvent control group. Compared with the solvent control group, the CCl_4_ group had significantly higher ALT, AST, ALP and γ-GT levels (all *p* < 0.001). Compared with the CCl_4_ group, the PHI groups (10, 20 and 40 mg/ kg) had significantly decreased ALT, AST, ALP and γ-GT levels (all *p* < 0.05) (Supplementary Material Serum biochemistry).

### Results of Collagen Deposition and Liver Biochemical Indexes

**FIGURE 3:**
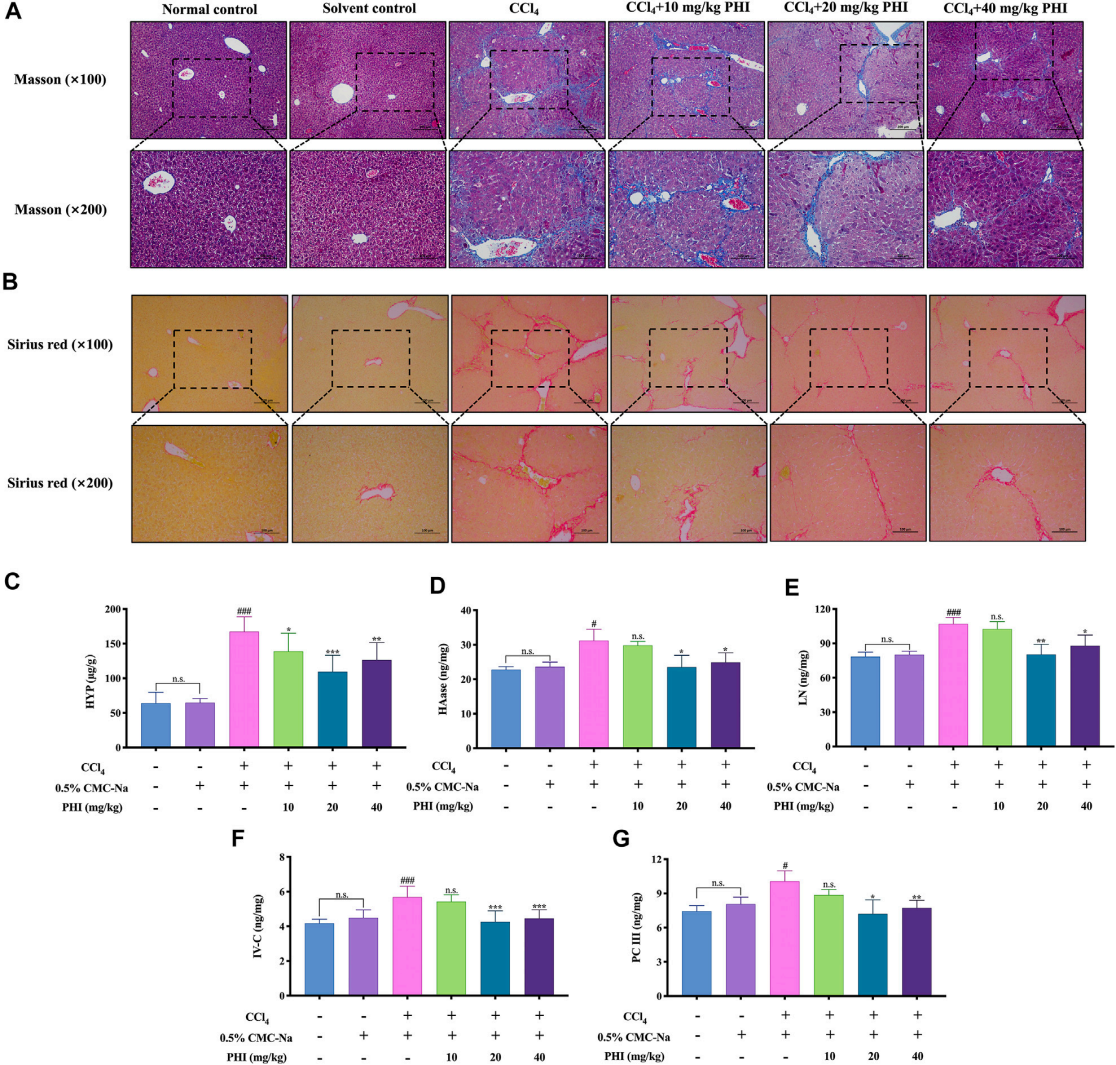
Effect of PHI on collagen deposition and liver biochemical indexes of liver fibrosis in mice. **(A)** Masson staining ( × 100 and × 200). **(B)** Sirius red staining ( × 100 and × 200). **(C)** Liver HYP activity. **(D)** Liver HAase activity. **(E)** Liver LN activity. **(F)** Liver IV-C activity. **(G)** Liver PC Ⅲ activity. Data was expressed as the mean ± SD (*n* = 6). ^#^ *p* < 0.05, ^###^ *p* < 0.001 represent compared with solvent control group. *p < 0.05, **p < 0.01, ***p < 0.001 represent compared with CCl_4_ group. n. s indicates no significant.

To observe the distribution of fibrous tissue, Masson staining and Sirius red staining were performed. As shown in Figures 3A,B, the collagen fibers were dyed blue in Masson staining and red in Sirius red staining. The results showed that there was almost no collagen fiber production in the normal control group and solvent control group, while CCl_4_ could induce the production of a large amount of collagen fibers. Interestingly, PHI (10, 20 and 40 mg/ kg) treatment markedly reduced collagen accumulation in the liver.

The results of liver biochemical indexes are shown in Figures 3C–G. There is no significant difference between the normal control group and the solvent control group. Compared with the solvent control group, the CCl_4_ group had significantly higher HYP, HAase, LN, IV-C and PC Ⅲ (all *p* < 0.05). Compared with the CCl_4_ group, the PHI groups (20 and 40 mg/kg) had significantly decreased HYP, HAase, LN, IV-C and PC Ⅲ levels (all *p* < 0.05) (Supplementary Material Liver biochemistry).

### PHI can Inhibit the Activation of Hepatic Stellate Cells

**FIGURE 4:**
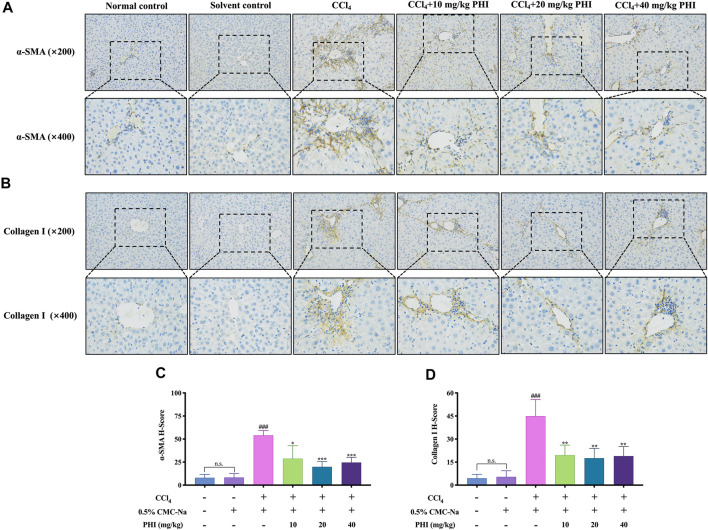
Effect of PHI on the expression of α-SMA and collagen I. **(A)** Immunohistochemistry staining of α-SMA in the liver tissues ( × 200 and × 400). **(B)** Immunohistochemistry staining of collagen I in the liver tissues ( × 200 and × 400). **(C)** Quantitative results of α-SMA in the liver tissues **(D)** Quantitative results of collagen I in the liver tissues. Data was expressed as the mean ± SD (*n* = 6). ^###^ *p* < 0.001 represent compared with solvent control group. *p < 0.05, **p < 0.01, ***p < 0.001 represent compared with CCl_4_ group. n. s indicates no significant.

Liver fibrosis is usually accompanied by HSCs activation, whereas α-SMA is the symbol of HSCs activation, and collagen I is one of the main components of ECM. To investigate the effect of PHI on HSCs activation, the expression level of α-SMA and collagen I were measured to determine whether PHI could inhibit HSCs activation against liver fibrosis. As shown in Figure 4, α-SMA and collagen I were dyed yellow. The results showed that α-SMA and collagen I were rarely observed in the normal control group and solvent control group, while CCl_4_ treatment significantly increased α-SMA and collagen I-positive cells in the liver. In contrast, α-SMA and collagen I immunoreactive cells were largely decreased by treatment with PHI (10, 20, and 40 mg/ kg) (Supplementary Material Immunohistochemistry).

### PHI can Inhibit Inflammation

**FIGURE 5:**
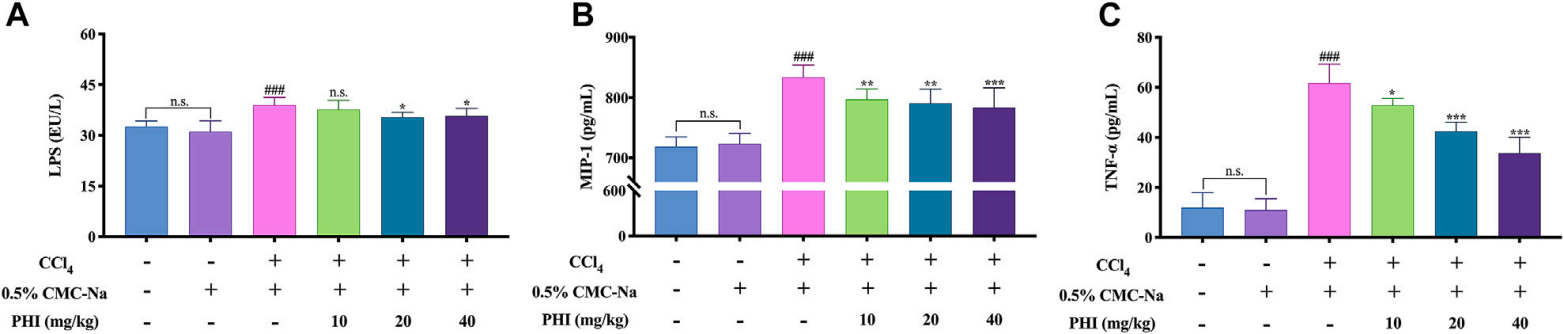
PHI inhibits inflammation. **(A)** Serum LPS level. **(B)** Serum MIP-1 level. **(C)** Serum TNF-α level. Data was expressed as the mean ± SD (*n* = 6). ^###^ *p* < 0.001 represent compared with solvent control group. *p < 0.05, **p < 0.01, ***p < 0.001 represent compared with CCl_4_ group. n. s indicates no significant.

To evaluate the anti-inflammatory effects of PHI, we detected the serum levels of LPS, MIP-1 and TNF-α. As shown in Figure 5, the levels of LPS, MIP-1 and TNF-α were not significantly different between the normal control group and the solvent control group. Compared with the solvent control group, the levels of LPS, MIP-1 and TNF-α in CCl_4_ group were significantly increased (all *p* < 0.001). On the contrary, the levels of LPS, MIP-1 and TNF-α were significantly reduced by the intervention of 20 and 40 mg/ kg PHI (all *p* < 0.05).

### PHI can Protect the Intestinal Epithelial Barrier Breakdown Induced by CCl_4_

**FIGURE 6:**
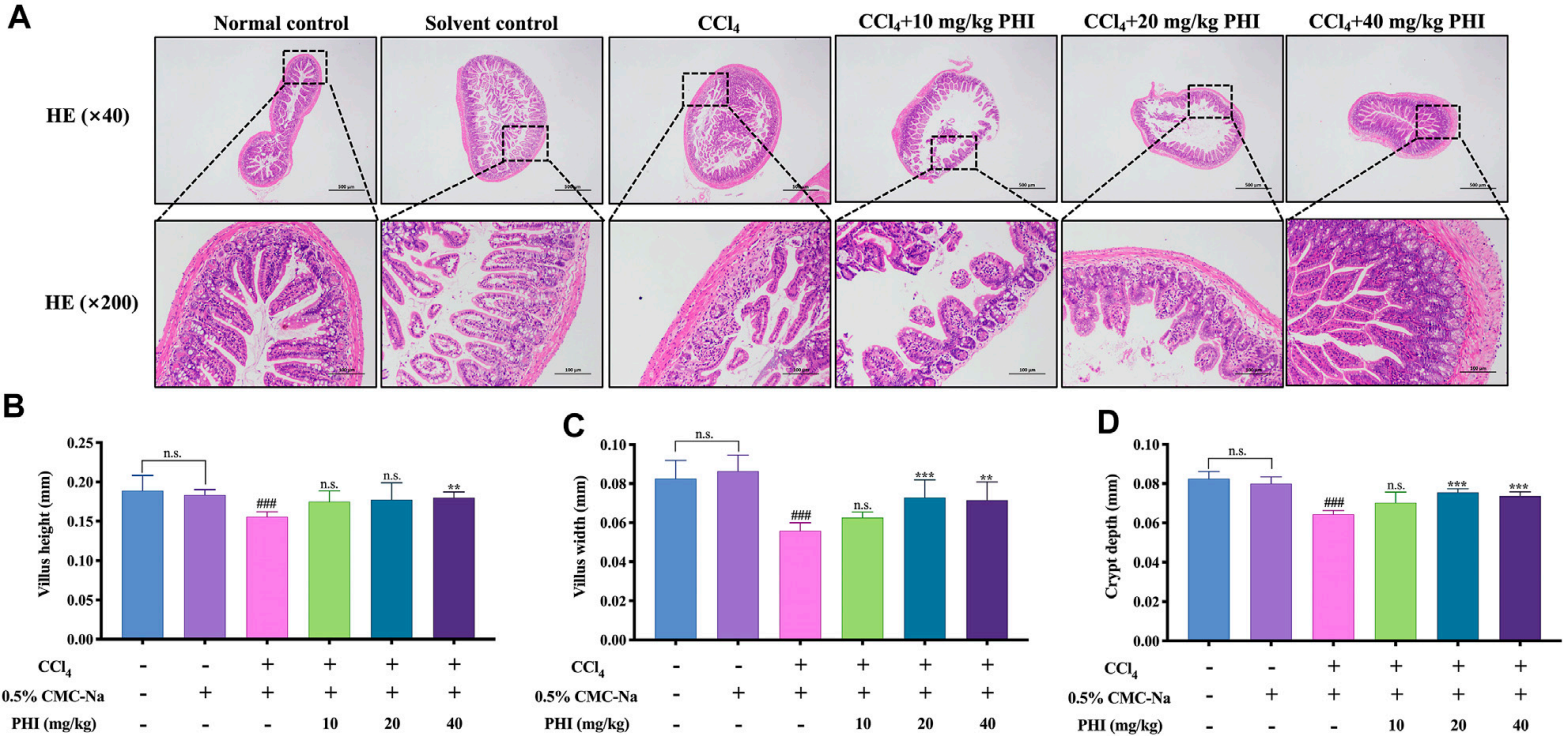
PHI protected intestinal epithelial barrier breakdown induced by CCl_4_. **(A)** HE staining of ileum ( × 40 and × 200) **(B)** Villus height. **(C)** Villus width. **(D)** Crypt depth. Data was expressed as the mean ± SD (*n* = 6). ^###^ *p* < 0.001 represent compared with solvent control group. **p < 0.01, ***p < 0.001 represent compared with CCl_4_ group. n. s indicates no significant.

As shown in Figure 6A, HE staining of ileum showed that the orderly uniformly distributed villi and intestinal glands were aligned in the ilea of the normal control group and the solvent control group. After CCl_4_ treatment of mice, a large number of intestinal gland epithelial cell degeneration, cell swelling, villi disorder and fragmentation were observed in the ileum. However, after PHI treatment, compared with the CCl_4_ group, the degeneration and swelling of intestinal gland epithelial cells and the infiltration of inflammatory cells in the ileum of mice in the PHI groups were alleviated to varying degrees, and the intestinal villi were more orderly and regular.

In addition, we also measured the villus height, villus width and crypt depth of the ileum of mice in each group, and the results are shown in Figures 6B–D. There is no significant difference between the normal control group and the solvent control group. Compared with the solvent control group, the villus height, villus width and crypt depth of the ileum in the CCl_4_ group were significantly reduced (all *p* < 0.001). However, the decrease of these indexes can be reversed after PHI (20 and 40 mg/ kg) treatment (Supplementary Material Iileum pathology). In conclusion, these results indicate that PHI can significantly improve the damaged intestine.

### PHI Changes the Overall Structure of Intestinal Microbiota

**FIGURE 7:**
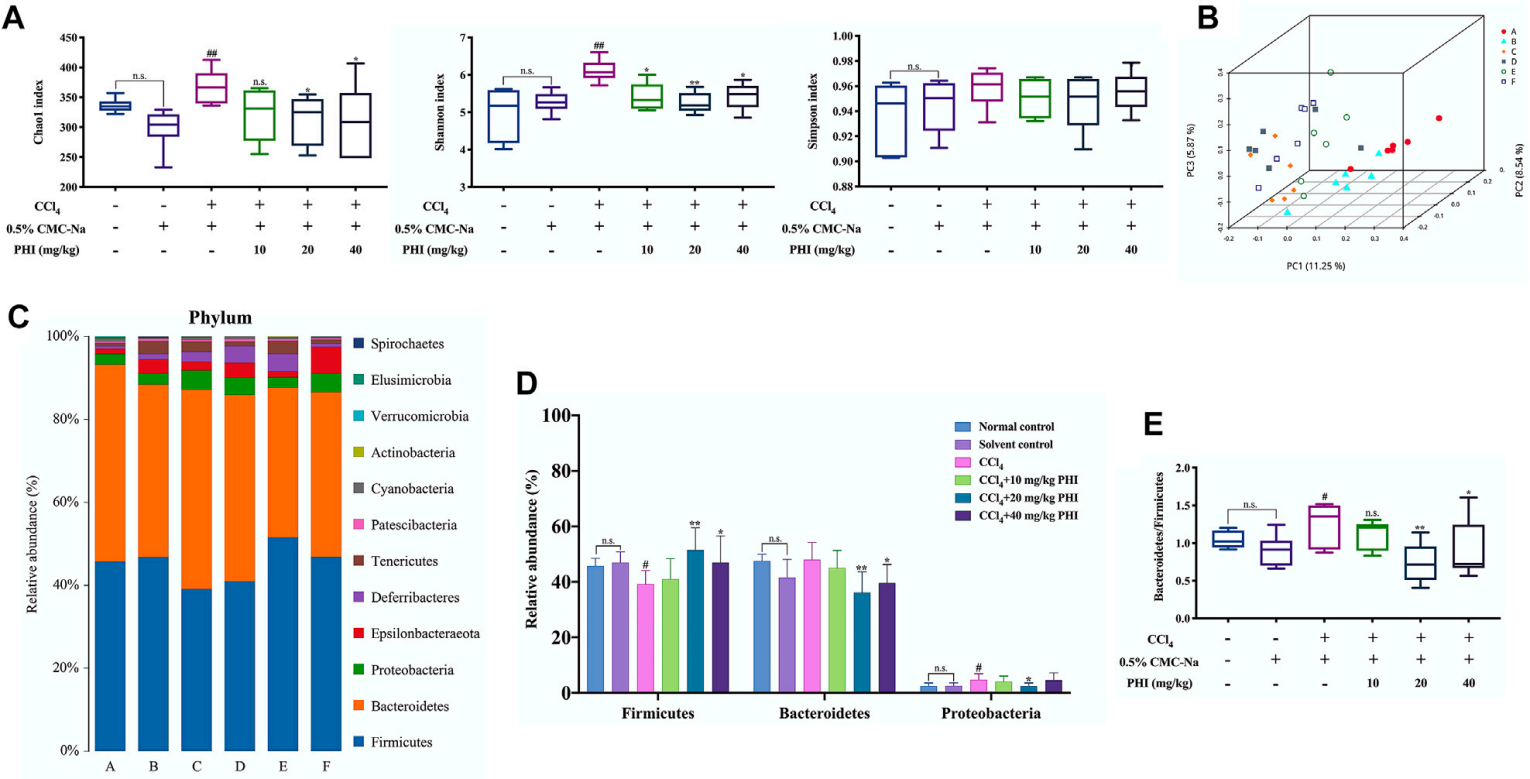
PHI modulates the composition of gut microbiota. **(A)** The alpha diversity of each group was obtained using the Chao1 index, Shannon index and Simpson index. **(B)** PCoA of gut microbiota. **(C)** The relative abundance of bacteria at the phylum level. **(D)** Representative histogram of the gut microbiota at the phylum level. **(E)** *Bacteroides* to Firmicutes ratio. A: Normal control; B: Solvent control; C: CCl_4_; D: CCl_4_ + 10 mg/kg PHI; E: CCl_4_ + 20 mg/ kg PHI; F: CCl_4_ + 40 mg/ kg PHI. Data was expressed as the mean ± SD (*n* = 6). ^#^ *p* < 0.05, ^##^ *p* < 0.01 represent compared with solvent control group. *p < 0.05, **p < 0.01 represent compared with CCl_4_ group. n. s indicates no significant.

After 36 samples were sequenced, a total of 270255 circular consensus sequencing sequences were obtained through Barcode identification, and each sample produced an average of 7507 circular consensus sequencing sequences. Using Usearch software to cluster Reads at a similarity level of 97.0%, a total of 682 OTUs were finally determined (Edgar, 2013). First of all, we employed Chao1, Shannon and Simpson indices to assess the richness, diversity, and evenness of gut microbiota (Figure 7A). The value of the Chao1 and Shannon indexes, were significantly higher in the CCl_4_ group than in the solvent control group (*p* < 0.01). Compared with the CCl_4_ group, PHI (20 and 40 mg/ kg) treatment significantly restored these indexes. Next, the principal coordinates analysis (PCoA) plot showed a shift in the overall gut microbiota between the solvent control group, CCl_4_ group, and the different concentrations of PHI group (Figure 7B). These results confirmed that the microbiota would change significantly in the process of liver fibrosis, and the corrective effects of PHI.

In addition, to access the effects of PHI on the faecal microbiota composition, we measured the composition of the microbial community in mice of all groups. At the phylum level, a significant decrease in Firmicutes and a significant increase in Proteobacteria were observed after CCl_4_ treatment (Figures 7C,D). PHI treatment significantly reversed the relative abundance of these gates (Figures 7C,D). Moreover, the ratio of *Bacteroides* to Firmicutes increased significantly in the CCl_4_ group, while decreased significantly in the PHI group (20 and 40 mg/ kg) (Figure 7E).

### PHI Ameliorates CCl_4_-Induced Intestinal Microbiota Disorder at Genus and Species Levels

**FIGURE 8:**
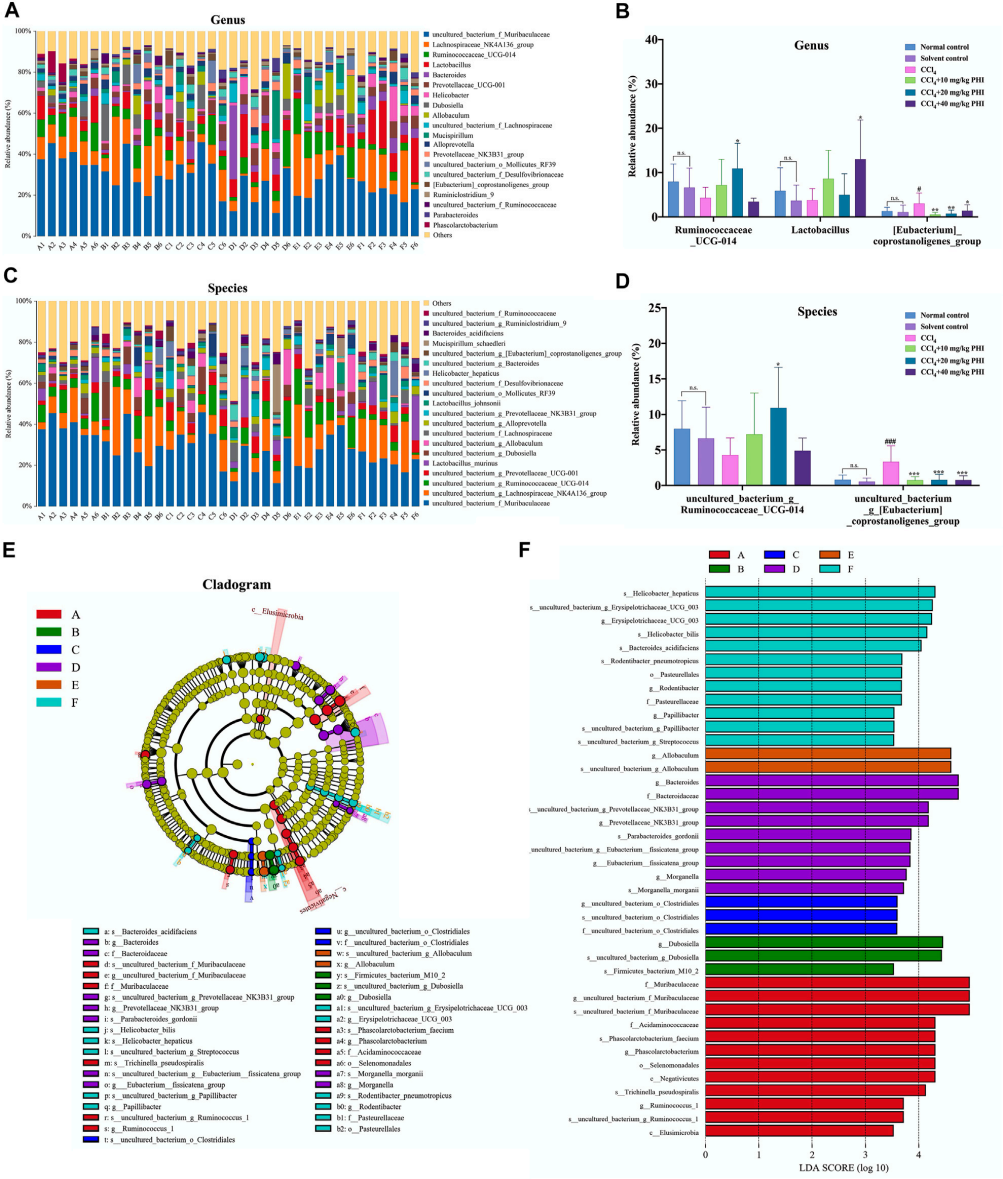
PHI modulates the composition of gut microbiota. **(A)** The relative abundance of the top 20 abundant bacteria at the genus level. **(B)** At the genus level, changes in microbiota in mice in each group. **(C)** The relative abundance of the top 20 abundant bacteria at the species level. **(D)** At the species level, changes in microbiota in mice in each group. **(E)** Linear discriminant analysis effect size (LEfSe) prediction was used to identify the most differentially abundant bacteria in each group. (F) LDA scores showed significant bacterial differences in each group. Only the bacteria meeting a significant LDA threshold value of 3.5 are shown. A: Normal control; B: Solvent control; C: CCl_4_; D: CCl_4_ + 10 mg/ kg PHI; E: CCl_4_ + 20 mg/ kg PHI; F: CCl_4_ + 40 mg/ kg PHI. Data was expressed as the mean ± SD (*n* = 6). ^#^ *p* < 0.05, ^###^ *p* < 0.001 represent compared with solvent control group. *p < 0.05, **p < 0.01, ***p < 0.001 represent compared with CCl_4_ group. n. s indicates no significant.

Subsequently, we studied the specific changes of intestinal microbiota at the genus level upon CCl_4_ and PHI treatment (Figure 8A). Detailed analysis at the genus level showed that CCl_4_ significantly increased the relative abundance of (*Eubacterium*)*_*coprostanoligenes_group (Figure 8B). Interestingly, the intervention of PHI reversed the abundance of this bacteria. In addition, PHI treatment significantly increased the abundance of *Ruminococcaceae_UCG-014* and *Lactobacillus* (Figure 8B). Similarly, at the species level, we found that the abundance of some bacteria was significantly changed upon CCl_4_ and PHI treatments (Figure 8C). In particular, CCl_4_ significantly increased the relative abundance of uncultured_bacterium_g*_(Eubacterium)_*coprostanoligenes_group (Figure 8D). However, PHI treatment at different concentrations reversed its abundance. Moreover, PHI treatment significantly increased the abundance of uncultured_bacterium_g_Ruminococcaceae_UCG-014 (Figure 8D).

Next, the linear discriminant analysis effect size (LEfSe) was used to analyze the composition of the microbiota, and similar results were obtained (Figure 8E). Our results indicated that there were 12, 3, 3, 9, 2, and 12 significant differences in the normal control, solvent control, CCl_4_, CCl_4_ + 10 mg/ kg PHI, CCl_4_ + 20 mg/ kg PHI and CCl_4_ + 40 mg/ kg PHI groups, respectively (Figure 8F).

Overall, these results further demonstrate that PHI can attenuate CCl_4_-induced liver fibrosis by regulating intestinal microbiota (Supplementary Material Gut microbiota).

### Function Prediction and Correlation Analysis

**FIGURE 9:**
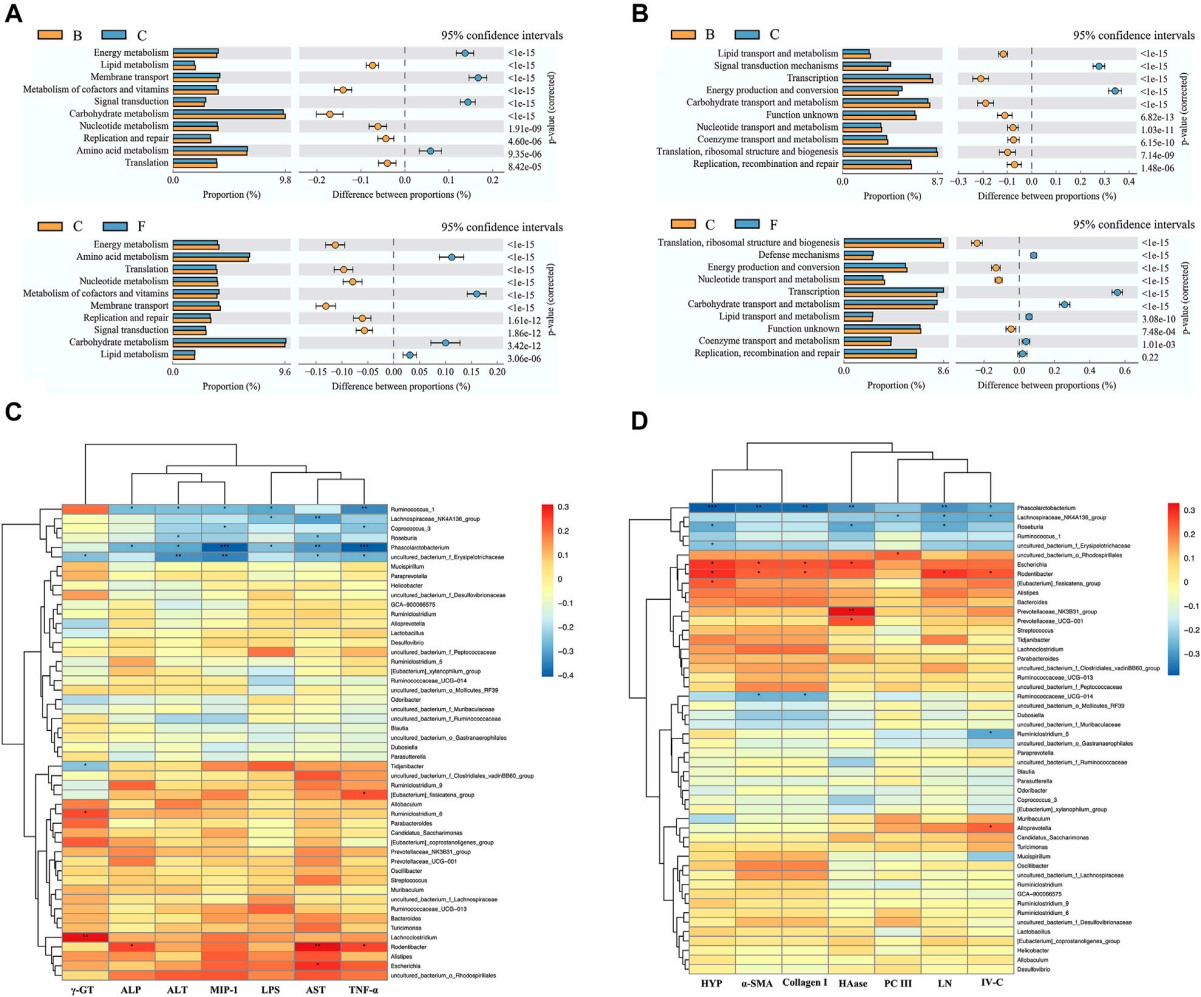
Function prediction analysis of gut microbiota and Correlation analysis heat map. **(A)** Prediction of microbiome function based on KEGG database. **(B)** Prediction of microbiome function based on COG database. **(C)** Correlation analysis of intestinal microflora and serum biochemical indexes in mice. **(D)** Correlation analysis of intestinal microflora and liver biochemical indexes in mice. B: Solvent control; C: CCl_4_; F: CCl_4_ + 40 mg/ kg PHI.

We predicted the function of the microbiome through the KEGG and COG databases (Figures 9A,B). The enriched pathways of the microbiome in CCl_4_-treated mice were as follows: “energy production and conversion”, “membrane transport” and “signal transduction mechanisms”. However, after liver fibrosis mice were treated with PHI (40 mg/ kg), the enriched pathways of the microbiome changed to the following pathways: “carbohydrate transport and metabolism”, “lipid transport and metabolism”, “metabolism of cofactors and vitamins”, “defense mechanisms”, “transcription” and “coenzyme transport and metabolism”.

Subsequently, we analyzed the correlation between serum biochemical indexes and intestinal flora species. As shown in Figure 9C, Phascolarctobacterium has a significant negative correlation with ALP (*p* < 0.05), ALT (*p* < 0.05), MIP-1 (*p* < 0.001), LPS (*p* < 0.05), AST (*p* < 0.01) and TNF-α (*p* < 0.001). Roseburia has a significant negative correlation with ALT (*p* < 0.05) and AST (*p* < 0.05). However, Rodentibacter has a significant positive correlation with ALP (*p* < 0.05), AST (*p* < 0.01) and TNF-α (*p* < 0.05). In addition, we also analyzed the correlation between liver biochemical indicators and intestinal flora species. As shown in Figure 9D, Phascolarctobacterium has a significant negative correlation with HYP (*p* < 0.001), α-SMA (*p* < 0.01), Collagen Ⅰ (*p* < 0.01), HAase (*p* < 0.01), LN (*p* < 0.01) and IV-C (*p* < 0.05). Roseburia has a significant negative correlation with HYP (*p* < 0.05), HAase (*p* < 0.05) and LN (*p* < 0.05). However, *Escherichia* has a significant positive correlation with HYP (*p* < 0.05), α-SMA (*p* < 0.05), Collagen Ⅰ (*p* < 0.05) and HAase (*p* < 0.05). Rodentibacter has a significant positive correlation with HYP (*p* < 0.05), α-SMA (*p* < 0.05), Collagen Ⅰ (*p* < 0.05), LN (*p* < 0.05) and IV-C (*p* < 0.05).

### RT-qPCR

**FIGURE 10:**
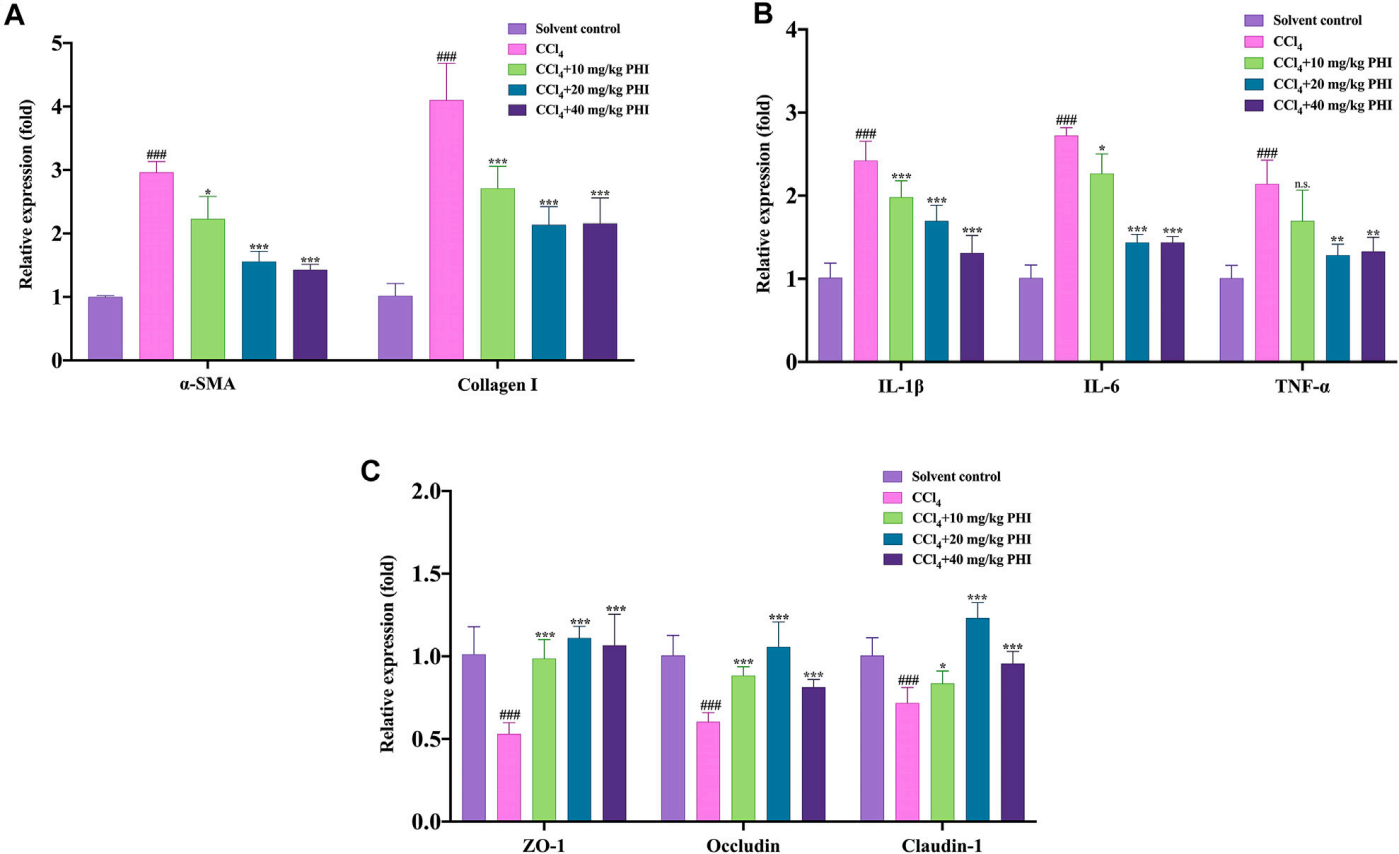
The relative mRNA expression levels in the solvent control, CCl_4_, CCl_4_ + 10 mg/ kg PHI, CCl_4_ + 20 mg/ kg PHI and CCl_4_ + 40 mg/ kg PHI groups. **(A)** The mRNA expressions of α-SMA and Collagen I. **(B)** The mRNA expressions of IL-1β, IL-6 and TNF-α. **(C)** The mRNA expressions of ZO-1, Occludin and Claudin-1. Data was expressed as the mean ± SD (n = 6). ^###^ *p* < 0.001 represent compared with solvent control group. *p < 0.05, **p < 0.01, ***p < 0.001 represent compared with CCl_4_ group. n. s indicates no significant.

First of all, we studied the expression of α-SMA and Collagen I mRNA. As shown in Figure 10A, the gene expression levels of α-SMA and Collagen I were significantly increased in the CCl_4_ group compared with the solvent control group. However, PHI (10, 20, and 40 mg/ kg) treatment reversed the abnormal expression of these two genes. Interestingly, these results are consistent with the results of immunohistochemistry staining. Subsequently, we studied the mRNA expression of three inflammatory factors, including IL-1β, IL-6, and TNF-α. As shown in Figure 10B, compared with the solvent control group, hepatic mRNA levels of IL-1β, IL-6 and TNF-α were remarkably increased in the CCl_4_ model group. In contrast, PHI (10, 20, and 40 mg/ kg) treatment significantly downregulated the expression of these genes.

Moreover, destruction of the intestinal barrier is an important intestinal change in liver fibrosis. Therefore, we use RT-qPCR to detect and quantify the mRNA levels of important markers of the intestinal barrier, including ZO-1, Occludin and Claudin-1. As shown in Figure 10C, the gene expression levels of ZO-1, Occludin and Claudin-1 were significantly decreased in the CCl_4_ group compared with the solvent control group. In fibrotic mice treated with PHI (10, 20, and 40 mg/ kg), the reduced level of ZO-1, Occludin and Claudin-1 mRNA expression was significantly restored (Supplementary Material RT-qPCR).

## Discussion

Liver fibrosis is a pathological feature of different underlying chronic liver disease exacerbations due to long-term liver injury, which is mainly characterized by excessive ECM accumulation (Böttcher and Pinzani, 2017; Tsuchida and Friedman, 2017). Currently, there are many factors causing liver fibrosis, including drug damage, excessive alcohol intake, infection with viruses, metabolic abnormalities, and autoimmune liver diseases (Henderson and Iredale, 2007; Kaffe et al., 2017). At the early stage, liver fibrosis is a reversible process (Sun and Kisseleva, 2015). On the contrary, if left untreated, ECM continuously accumulates to form a fibrous scar, which in turn progresses largely to irreversible cirrhosis and even hepatocellular carcinoma (Jung and Yim, 2017). Unfortunately, despite the tremendous progress has been made in the study of the pathogenesis associated with liver fibrosis, there is no specific and effective method to prevent the development of liver fibrosis in the clinic (Schuppan and Kim, 2013). Therefore, there is an urgent need to find satisfactory therapeutic drugs for liver fibrosis.

PHI, a lignan isolated from Forsythia suspensa, has been reported to have many biological functions, including anti-inflammation (Hu et al., 2020), antioxidation (Chang et al., 2008), anti-allergy (Sung et al., 2016), and hepatoprotective (Song et al., 2018). To deeply explore the therapeutic effects of PHI on liver fibrosis and the underlying mechanisms, we established a CCl_4_-induced liver fibrosis model in mice. The model has been commonly used to study the mechanism of action of liver fibrosis worldwide, and it is also the closest model to human liver fibrosis (Scholten et al., 2015). At the end of the experiment, we first performed histological analysis including HE, Masson, and Sirius red staining of the livers from mice in each group, as liver tissue injury is an initiating factor in the development of liver fibrosis. The hepatic histopathological changes in mice from the CCl_4_ group were successfully observed by HE staining, which specifically showed hepatocyte steatosis, eosinophilic cytoplasm enhancement, lymphocyte infiltration and collagen fiber hyperplasia. Similarly, the production of a large number of collagen fibers in the livers of mice in the CCl_4_ group was also observed by Masson staining and Sirius red staining. This suggested that the CCl_4_-induced liver fibrosis model in mice was successfully established. However, after the treatment with PHI (10, 20, and 40 mg/ kg), the above mentioned hepatic pathological changes could be ameliorated to various degrees, which indicated that PHI exerted a good alleviating effect on CCl_4_-induced liver fibrosis.

At present, ALT and AST are the most commonly used indicators for the clinical diagnosis of hepatocellular injury. When lesions occur in the liver, such as destruction of the hepatocyte membrane by CCl_4_, the two enzymes will be released into the extracellular space and enter the systemic circulation, thereby significantly increasing their serum concentrations (Ozer et al., 2008). In addition, AKP and γ-GT are also able to directly reflect the liver tissue damage and are important for the differential and diagnosis of liver diseases (Liu et al., 2019). Our results showed that PHI significantly reduced the serum levels of AST, ALT, AKP and γ-GT, which indicated that PHI could effectively ameliorate CCl_4_-induced liver injury. In addition, to further explore the anti-fibrotic effect of PHI, we investigated the effect of PHI on collagen synthesis and deposition. HYP is one of the major components of collagenous tissues. When connective tissues in the body are greatly proliferated or destroyed, such as liver fibrosis and cirrhosis, HYP content in the blood and tissues increases. Therefore, the measurement of the content of HYP becomes an important measure of the metabolism of collagen tissue *in vivo* (Bolarin and Azinge, 2007). In addition, the four tests of liver fiber, including HAase, LN, IV-C and PC III, are important to measure the activity of inflammation as well as the degree of fibrosis in chronic liver diseases (Younesi and Parsian, 2019). Our data showed that the expression of HYP, HAase, LN, IV-C and PC III in the livers of mice in the CCl_4_ group was significantly increased. Compared with the CCl_4_ group, the PHI groups (20 and 40 mg/ kg) significantly decreased the levels of HYP, HAase, LN, IV-C, and PC III. These results were consistent with the results of histopathological assays, which reconfirmed that PHI could effectively ameliorate CCl_4_-induced liver fibrosis.

The activation of HSCs is well recognized as a central event during the initiation phase of liver fibrosis (Dhar et al., 2020). Meanwhile, HSCs activation is also a major source of ECM production, including α-SMA, Collagen I, and so on (Kisseleva and Brenner, 2021). Among them, α-SMA is a major marker of HSCs activation (Jiao et al., 2009). Therefore, inhibiting the proliferation and activation of HSCs is the key to preventing liver fibrosis. Correspondingly, we investigated the expressions of α-SMA and Collagen I. Immunostaining showed that the α-SMA and Collagen I expressions were significantly higher in the CCl_4_ group than the solvent control group. Our results were in agreement with the findings of Yan et al. (Yan et al., 2021). This indicates that CCl_4_ is able to promote the activation of HSCs as well as the accumulation of ECM. Interestingly, after PHI treatment, the expressions of α-SMA and Collagen I were significantly decreased. Similarly, the subsequent detection results of α-SMA and Collagen I mRNA expressions were consistent with this. This suggests that PHI reduced ECM production, which in turn alleviated liver fibrosis, possibly by inhibiting the activation of HSCs.

In recent years, intestinal flora has become a research hotspot. Within the human gut, there are more than a thousand bacterial species, totaling approximately 100 trillion, and Bacteroidetes and Firmicutes account for more than 90% of the total bacterial population (Chopyk and Grakoui, 2020). Under normal conditions, the proportion and quantity of each flora in the body are relatively stable. However, when the internal and external environment of the host is changed, such as improper diet, drug abuse, etc., the intestinal flora *in vivo* may be disordered, which is mainly manifested by the decrease of beneficial bacteria and the increase of harmful bacteria (Leung et al., 2016). What’s more, at present, a large number of studies have shown that intestinal flora imbalance is closely related to the occurrence and development of liver fibrosis (Yu and Schwabe, 2017; Zhou et al., 2019). In view of the important role of gut microbiota in the development of liver fibrosis, this study employed 16 S rRNA gene sequencing to explore whether the beneficial effects of PHI partly depend on the regulation of intestinal flora imbalance. Our data demonstrated a significant modulation of the gut microbiota by PHI. At the phylum level, the number of Firmicutes, a bacterial phylum proven to be beneficial to the body, was significantly decreased in the CCl_4_ group compared to the solvent control group. Our results were also in agreement with those of Wan et al. (Wan et al., 2019). Interestingly, PHI treatment significantly reversed the relative abundance of Firmicutes. In addition, the comparison of the gut microbial structure between the CCl_4_ group and the PHI groups showed that there was also a decreasing trend in the ratio of Bacteroidetes to Firmicutes, which was consistent with the study by Zhang et al. (Zhang J. et al., 2018). At the genus level, we observed that PHI treatment enriched the relative abundance of Ruminococcaceae_UCG-014 and Lactobacillus, and decreased that of [*Eubacterium*]*_*coprostanoligenes_group. Among them, *lactobacillus* has the ability to protect the liver, and can attenuate inflammation and fibrosis in the injured liver (Jantararussamee et al., 2021). Collectively, these results further demonstrated that PHI could attenuate CCl_4_-induced liver fibrosis by modulating gut microbiota.

When gut microbiota is disrupted or dysbiosis occurs, a large number of bacteria grow in the gut and produce many metabolites, such as LPS, an endotoxin produced by Gram negative bacteria, can cause an increase in intestinal permeability (Stevens et al., 2018; Cardinale et al., 2020). Subsequently, some intestinal harmful bacteria and their components and metabolites enter the blood and liver through the portal venous system, and then activate the systemic immune response, which in turn activate macrophages and promote the release of many inflammatory cytokines, ultimately accelerating the development of liver fibrosis (Kumar et al., 2011; Zhou et al., 2019). Our data showed that PHI had a good anti-inflammatory effect in mice with CCl_4_-induced liver fibrosis, which represented as the reduction of serum LPS, MIP-1 and TNF-α levels as well as the decreased expressions of three inflammatory factors (IL-1β, IL-6, and TNF-α) in liver tissue.

It is well known that intestinal flora disorder also disrupts the integrity of tight junctions in the intestinal epithelium, leading to intestinal barrier dysfunction (Winer et al., 2016). Our results showed that massive intestinal glandular epithelial cell degeneration, cell swelling, villus disorganization, and fragmentation occur in the ileum of mice after CCl_4_ treatment, which is consistent with the conclusions of the studies by Wan et al. (Wan et al., 2019) and Yan et al. (Yan et al., 2019). However, after PHI treatment, the pathological conditions of ileum were all alleviated to various degrees, and the intestinal villi were neater and more regular. Similarly, the determination of some ileal indices, including villus height, villus width, and crypt depth, also confirmed that PHI could significantly improve the damaged intestine. More importantly, tight junction proteins are the markers of intestinal barrier integrity, and are important for maintaining intestinal integrity (Zeisel et al., 2019). Therefore, we also examined the important markers of intestinal barrier, including ZO-1, Occludin, and Claudin-1. The results showed that CCl_4_ significantly decreased the expression of these genes, whereas PHI (10, 20 and 40 mg/ kg) significantly restored the expression levels of ZO-1, Occludin, and Claudin-1. To sum up, PHI can protect against CCl_4_-induced intestinal epithelial barrier disruption.

**FIGURE 11:**
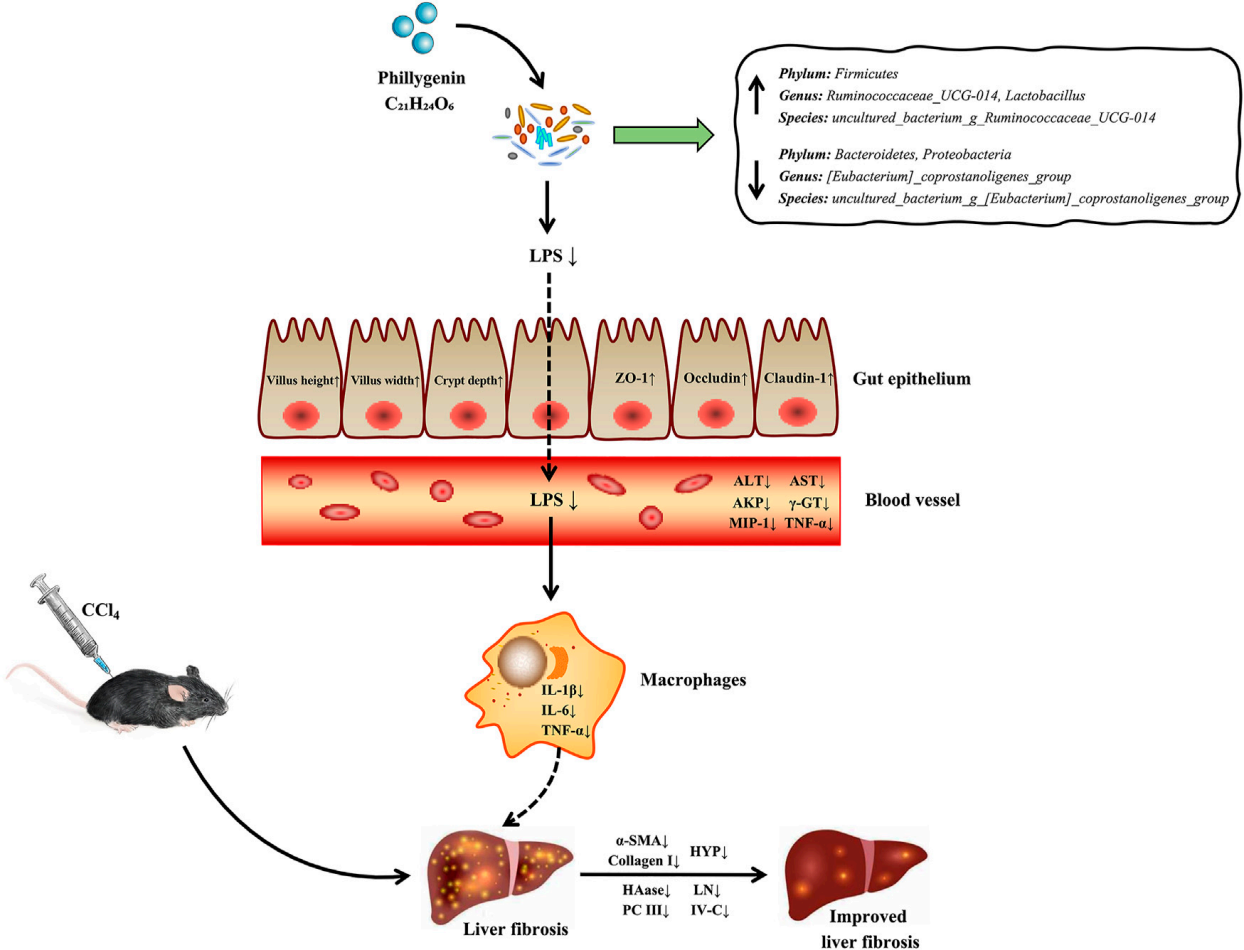
The potential mechanism of PHI in alleviating CCl_4_-induced liver fibrosis.

Given the above, we proposed a possible mechanism of PHI alleviates CCl_4_-triggered liver fibrosis (Figure 11). PHI administration attenuates CCl_4_-induced liver fibrosis by regulating inflammation and gut microbiota.

## Conclusion

In conclusion, our study clearly demonstrated that PHI could improve liver histopathological injury, abnormal liver function, collagen deposition, endotoxemia and fibrosis caused by CCl_4_. Moreover, the molecular mechanism of PHI’s hepatoprotective effect involved modulating inflammation and gut microbiota according to 16 S rRNA gene sequencing and molecular biology analysis. This study may provide a potential novel idea for the treatment strategy of PHI in CCl_4_-induced liver fibrosis.

## Data Availability

The data presented in the study are deposited in the Sequence Read Archive repository, accession number PRJNA758124; Our data has been released.

## Abbreviations

ALP, alkaline phosphatase; ALT, alanine aminotransferase; AST, aspartate aminotransferase; CCl4, carbon tetrachloride; CMC-Na, sodium carboxymethyl cellulose; IV-C, collagen type Ⅳ; ECM, extracellular matrix; ELISA, enzyme-linked immunosorbent assay; H and E, Hematoxylin and eosin; HAase, hyaluronic acid; HSCs, hepatic stellate cells; HYP, hydroxyproline; IL-1β, interleukin-1 beta; IL-6, interleukin-6; LN, laminin; LPS, lipopolysaccharide; MIP-1, macrophage inflammatory protein 1; PCoA, principal coordinates analysis; PCoA, principal coordinates analysis; PHI, phillygenin; RT-qPCR, real-time quantitative PCR; α-SMA, α-smooth muscle actin; PC Ⅲ, precollagen type Ⅲ; TNF-α, tumor necrosis factor α; γ-GT, γ-glutamyl transpeptidase; LEfSe, linear discriminant analysis effect size.
