# Supplementary material for: Phillygenin Attenuates Carbon Tetrachloride-Induced Liver Fibrosis via Modulating Inflammation and Gut Microbiota
Source: Front Pharmacol. 2021 Sep 21;12:756924. doi: 10.3389/fphar.2021.756924 (PMC8490881; doi:10.3389/fphar.2021.756924)
Supplement: Supplementary file 1 [file DataSheet1.ZIP › Source data/Link.docx]

All original microscope images in the article are stored in the following link.

Link: <https://www.jianguoyun.com/p/DTViQ0sQwNvgCRiQnIcE>

Access password: Ck5sQQ
